# Comparison of the Cytotoxic Mechanisms of Different Garlic (*Allium sativum* L.) Cultivars with the Crucial Involvement of Peroxisome Proliferator-Activated Receptor Gamma

**DOI:** 10.3390/ijms26010387

**Published:** 2025-01-04

**Authors:** Urszula E. Binduga, Aneta Kopeć, Joanna Skoczylas, Konrad A. Szychowski

**Affiliations:** 1 Department of Civilization Diseases and Regenerative Medicine, Medical College, University of Information Technology and Management in Rzeszów, St. Sucharskiego 2, 35-225 Rzeszów, Poland; 2 Department of Human Nutrition and Dietetics, Faculty of Food Technology, Agricultural University of Krakow, St. Balicka 122, 30-149 Kraków, Poland; aneta.kopec@urk.edu.pl (A.K.); joannaskoczylas7@gmail.com (J.S.); 3 Department of Biotechnology and Cell Biology, Medical College, University of Information Technology and Management in Rzeszów, St. Sucharskiego 2, 35-225 Rzeszów, Poland; kszychowski@wsiz.edu.pl

**Keywords:** *Allium sativum* L., toxicity, PPARγ, apoptosis, catechin

## Abstract

Garlic (*Allium sativum* L.) is one of the oldest known useful plants, valued for thousands of years. This plant contains many biologically active compounds, including polyphenols, sterols, cysteine-sulfoxides, carbohydrates, proteins, and amino acids. The aim of our study was to compare the antioxidant potential, cytotoxicity, and apoptosis induction properties of four garlic cultivars—Harnaś, Ornak, Violeta, and Morado—in human squamous carcinoma (SCC-15) cells, colon adenocarcinoma (CACO-2) cells, and normal fibroblasts (BJ). Additionally, we investigated the mRNA and protein expression of peroxisome proliferator-activated receptor gamma (PPARγ), microtubule-associated protein 1 light chain 3 (LC3A), superoxide dismutase 1 (SOD1), and catalase (CAT) after treatment with the studied garlic extracts. Our study demonstrated that high ROS production was correlated with the strong toxicity of the garlic extracts. All studied extracts produced a lesser increase in ROS in normal BJ fibroblasts and were less toxic to these cells. The expression patterns of PPARγ, LC3A, SOD1, and CAT, along with chromatographic analysis, suggest differing mechanisms among the garlic cultivars. The highest levels of catechin, a known PPARγ agonist, were detected in the Harnaś (3.892 µg/mL) and Ornak (3.189 µg/mL) cultivars. A high catechin content was correlated with similar changes in PPARγ and related SOD1 and LC3A. Our findings showed the health-promoting and anticancer properties of garlic. However, we could not definitively identify which polyphenol or how it is involved in PPARγ activation. Further studies are required to elucidate the role of PPARγ in the mechanism of action of garlic extracts.

## 1. Introduction

Garlic (*Allium sativum* L.) is one of the oldest plants known to man, having been used for thousands of years. Currently, it is cultivated in most countries within the temperate zone [1]. Garlic, a member of the Amaryllidaceae family, is included in the group of natural food products known as ‘superfoods’. This term reflects not only its value as a spice but also its wide range of health-promoting effects [2]. Garlic contains various minerals, including vitamins C and B6, as well as numerous biologically active substances [3]. Its health benefits result from bioactive compounds such as polyphenols, sterols, cysteine sulfoxides, carbohydrates, proteins, and amino acids [4]. These compounds are responsible for garlic’s potent antibacterial properties against both Gram-positive and Gram-negative bacteria, including antibiotic-resistant strains [5]. Garlic also exhibits antiviral [6], antifungal [7], and, according to some studies, selective cytotoxic properties against cancer cell lines [8]. Moreover, recent studies show that garlic extracts reduced the progression of tumours in animal models and suppressed cancer cell growth [9].

Reactive oxygen species (ROS) are natural by-products of cellular oxidative metabolism [10]. In normal cells, ROS have numerous physiological and pathological functions. However, cancer cells possess a fragile ROS balance and are highly sensitive to disturbances, a phenomenon utilised in cancer therapeutic strategies [11]. Garlic polyphenols are a major group of substances responsible for the antioxidant and health-promoting properties of this plant [12]. In normal cells, garlic polyphenols can directly affect ROS levels or indirectly influence the production of antioxidant enzymes, resulting in long-term beneficial effects [13]. Conversely, in cancerous cells, garlic extracts have been shown to induce toxicity through ROS-dependent pathways [14].

Peroxisome proliferator-activated receptor gamma (PPARγ) is a master regulator of adipogenic differentiation and cell metabolism [15]. However, PPARγ and its ligands are also involved in cell differentiation, autophagy, antioxidant enzyme production, and apoptotic cell death [16]. Numerous studies have shown that PPARγ is essential for the proper activation of inflammatory processes and the regulation of antioxidant enzymes such as superoxide dismutase 1 (SOD1) and catalase (CAT) [16,17]. Moreover, both SOD1 and CAT are reportedly influenced by garlic extracts [17,18]. Similarly, reports suggest that biologically active compounds in garlic induce autophagic cell death involving LC3 protein in the human liver cancer (HepG2) cell line [19], a process that can also be partially regulated by PPARγ. To date, only a few studies have indicated that aged garlic extract, alliin (a garlic organosulfur compound), or garlic-derived diallyl disulfide may affect PPARγ molecular pathways in different cell culture models [20,21,22]. However, there is a lack of studies examining the role of PPARγ in apoptosis and toxicity after exposure of cancer cell lines to whole garlic extract, as well as comparisons with normal cell lines as controls. According to literature data, polyphenols that can be detected in garlic, such as, inter alia, catechin, quercetin, and kaempferol, can be full or partial PPARγ agonists. Therefore, it is likely that the tested extracts will activate the mentioned receptor.

The aim of our study was to compare the antioxidant potential, cytotoxicity, and apoptosis-inducing properties of four garlic cultivars in the human squamous carcinoma (SCC-15) cell line, colon adenocarcinoma (CACO-2) cell line, and normal fibroblast (BJ) cell line. Due to different growth and climate conditions, in our study the two garlic cultivars from Poland (Harnaś, Ornak) and two garlic cultivars from Spain (Violeta and Morado) were selected for analysis. We also evaluated the expression of selected genes and proteins associated with cell metabolism, free radical neutralisation, and the processes of autophagy and apoptosis.

## 2. Results

### 2.1. ROS Levels in Studied Cell Lines

**Figure 1:**
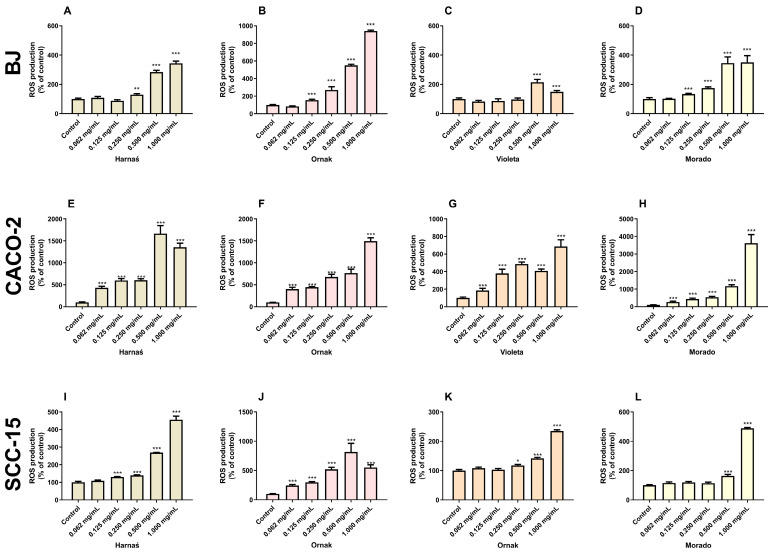
Effect of increasing concentrations (0.062, 0.125, 0.250, 0.500, and 1.000 mg/mL) of extracts from garlic cultivars Harnaś, Ornak, Violeta, and Morado on ROS production in (**A**–**D**) BJ, (**E**–**H**) CACO-2, and (**I**–**L**) SCC-15 cells after 24 h of exposure. Results are presented as mean ± SD. Statistically significant differences from the control are marked as * *p* < 0.05, ** *p* < 0.01, and *** *p* < 0.001.

Our results showed that the Harnaś garlic extract significantly increased ROS levels in BJ cells at concentrations of 0.250, 0.500, and 1.000 mg/mL, with increases of 29.8%, 184.8%, and 243.6%, respectively, compared with the control (Figure 1A). The strongest increase in ROS levels following Harnaś cultivar treatment was observed in CACO-2 cells at all tested concentrations (0.062, 0.125, 0.250, 0.500, and 1.000 mg/mL), with significant increases of 330.5%, 498.5%, 505.3%, 1566.2%, and 1255.7%, respectively, compared with the control (Figure 1E). In SCC-15 cells, a significant increase in ROS levels was observed at concentrations of 0.125, 0.250, 0.500, and 1.000 mg/mL, although it was not as pronounced as in the CACO-2 line, with increases of 30.6%, 40.0%, 168.9%, and 355.8%, respectively, compared with the control (Figure 1I).

In the BJ cell line, the Ornak garlic cultivar at concentrations of 0.125, 0.250, 0.500, and 1.000 mg/mL induced a significant increase in ROS production relative to the control group by 54.2%, 170.2%, 450.4%, and 841.2%, respectively (Figure 1B). In the CACO-2 cell line, the Ornak garlic cultivar significantly increased ROS production at concentrations of 0.062, 0.125, 0.250, 0.500, and 1.000 mg/mL by 302.4%, 343.5%, 677.6%, 666.3%, and 1393.4%, respectively, compared with the control group (Figure 1F). A similar significant increase in ROS levels was observed in the SCC-15 cell line across the concentration range of 0.062 to 1.000 mg/mL, with increases of 144.8%, 197.3%, 418.9%, 715.5%, and 450.1%, respectively, compared with the control group (Figure 1J).

The Violeta garlic cultivar significantly increased ROS levels in the BJ cell line only at the two highest concentrations (0.500 and 1.000 mg/mL), with increases of 114.5% and 49.3%, respectively, compared with the control group (Figure 1C). In the CACO-2 cell line, the Violeta cultivar enhanced ROS production across the concentration range of 0.062 to 1.000 mg/mL, with significant increases of 85.9%, 279.3%, 387.5%, 308.6%, and 586.3%, respectively, compared with the control group (Figure 1G). In the SCC-15 cell line, the Violeta garlic extract increased ROS production at concentrations of 0.250, 0.500, and 1.000 mg/mL, with significant increases of 41.5%, 135.0%, and 387.5%, respectively, compared with the control group (Figure 1K).

The extract of the Morado garlic cultivar enhanced ROS production in BJ cells at concentrations of 0.125, 0.250, 0.500, and 1.000 mg/mL, with significant increases of 34.5%, 75.2%, 245.2%, and 250.6%, respectively, compared with the control (Figure 1D). In the CACO-2 cell line, the extract caused a significant increase in ROS production at all tested concentrations (0.062 to 1.000 mg/mL), with increases of 166.3%, 337.1%, 439.1%, 1067.0%, and 33520.3%, respectively, compared with the control group (Figure 1H). In the SCC-15 cell line, the Morado cultivar extract significantly increased ROS production at the highest concentrations of 0.500 and 1.000 mg/mL, with increases of 64.3% and 388.8%, respectively, compared with the control group (Figure 1L).

### 2.2. Level of LDH Released

**Figure 2:**
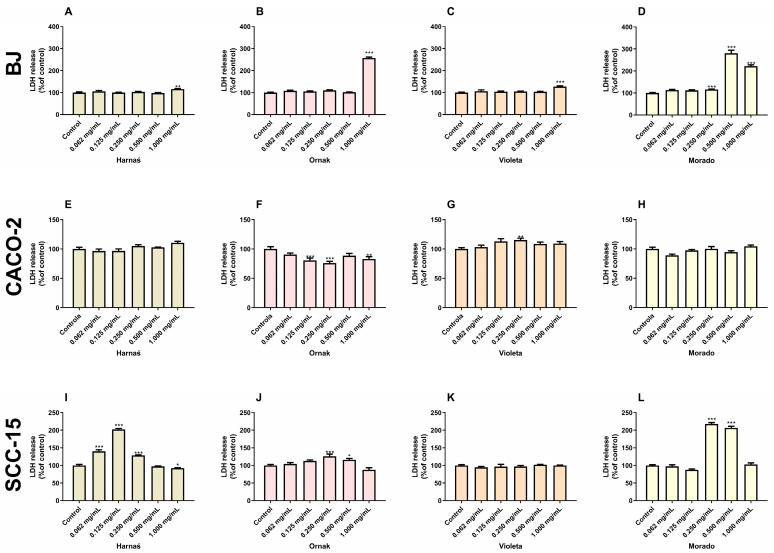
Effect of increasing concentrations (0.062, 0.125, 0.250, 0.500, and 1.000 mg/mL) of extracts from garlic cultivars Harnaś, Ornak, Violeta, and Morado on LDH release in (**A**–**D**) BJ, (**E**–**H**) CACO-2, and (**I**–**L**) SCC-15 cells after 24 h of exposure. Results are presented as mean ± SD. Statistically significant differences from the control are marked as * *p* < 0.05, ** *p* < 0.01, and *** *p* < 0.001.

The experiments showed that the Harnaś garlic cultivar significantly increased LDH release from BJ cells only at the highest concentration (1.000 mg/mL), with an increase of 16.0% compared with the control (Figure 2A). Interestingly, in the CACO-2 cell line, this garlic variety did not enhance LDH release at any tested concentration (Figure 2E). In the SCC-15 cell line, the Harnaś cultivar significantly increased LDH release at concentrations of 0.062, 0.125, and 0.250 mg/mL (40.2%, 102.3%, and 28.4%, respectively), while at 1.000 mg/mL, a decrease in LDH release was observed (7.7%) compared with the control (Figure 2I).

For the Ornak garlic cultivar, LDH release in BJ cells increased only at the highest concentration (1.000 mg/mL), with a rise of 157.1% compared with the control (Figure 2B). Interestingly, in the CACO-2 cell line, the tested garlic extract caused a significant decrease in LDH release at concentrations of 0.125, 0.250, and 1.000 mg/mL, with reductions of 19.63%, 23.9%, and 17.7%, respectively, compared with the control (Figure 2F). In the SCC-15 cell line, the extract significantly increased LDH release at concentrations of 0.250 and 0.500 mg/mL, with rises of 25.9% and 16.1%, respectively, compared with the control (Figure 2J).

The Violeta garlic cultivar, like the Harnaś and Ornak cultivars, significantly increased LDH release in the BJ cell line only at the highest concentration (1.000 mg/mL), with an increase of 27.6% compared with the control group (Figure 2C). In the CACO-2 cell line, a concentration of 0.250 mg/mL increased LDH release by 15.3% compared with the control (Figure 2G). The Violeta garlic extract did not alter LDH release in SCC-15 cells (Figure 2K).

For the Morado garlic cultivar extract in the BJ cell line, a significant increase in LDH release was observed at concentrations of 0.250, 0.500, and 1.000 mg/mL, with increases of 75.2%, 245.2%, and 250.6%, respectively, compared with the control (Figure 2D). In the CACO-2 cell line, no changes in LDH release were observed following administration of the Morado extract (Figure 2H). In the SCC-15 cell line, the Morado extract at a concentration of 0.125 mg/mL reduced LDH release by 12.3% compared with the control group, while concentrations of 0.250 and 0.500 mg/mL increased LDH release by 117.7% and 106.6%, respectively, compared with the control (Figure 2L).

### 2.3. Resazurin Reduction Assay

**Figure 3:**
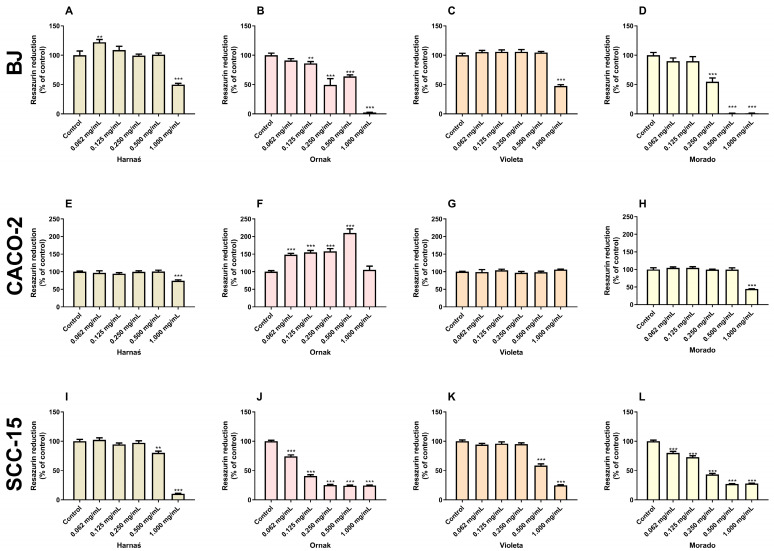
Effect of increasing concentrations (0.062, 0.125, 0.250, 0.500, and 1.000 mg/mL) of extracts from garlic cultivars Harnaś, Ornak, Violeta, and Morado on the level of resazurin reduction in (**A**–**D**) BJ, (**E**–**H**) CACO-2, and (**I**–**L**) SCC-15 cells after 24 h of exposure. Results are presented as mean ± SD. Statistically significant differences from the control are marked as ** *p* < 0.01 and *** *p* < 0.001.

The resazurin reduction test showed that the Harnaś garlic cultivar extract significantly increased resazurin reduction in BJ cells at a concentration of 0.062 mg/mL by 22.0% compared with the control group. However, at the highest concentration (1.000 mg/mL), a decrease in resazurin reduction of 50.2% was observed compared with the control group (Figure 3A). In CACO-2 cells, only the highest concentration of the Harnaś garlic extract (1.000 mg/mL) significantly reduced resazurin reduction by 25.9% compared with the control (Figure 3E). In SCC-15 cells, the Harnaś garlic extract reduced resazurin reduction at concentrations of 0.500 and 1.000 mg/mL by 19.7% and 89.6%, respectively, compared with the control (Figure 3I).

Garlic extract of the Ornak cultivar significantly decreased the level of resazurin reduction in the BJ cell line at concentrations of 0.125, 0.250, 0.500, and 1.000 mg/mL by 14.0%, 50.6%, 36.0%, and 97.8%, respectively, compared with the control (Figure 3B). Interestingly, in the CACO-2 cell line, the Ornak garlic extract at concentrations of 0.062, 0.125, 0.250, and 0.500 mg/mL increased the level of resazurin reduction by 48.2%, 55.2%, 58.1%, and 109.9%, respectively, compared with the control group (Figure 3F). In the SCC-15 cell line, all tested concentrations (0.062, 0.125, 0.250, 0.500, and 1.000 mg/mL) of the Ornak garlic extract caused a decrease in resazurin reduction by 25.2%, 59.4%, 74.8%, 75.8%, and 75.4%, respectively, compared with the control (Figure 3J).

Our experiments show that the Violeta garlic cultivar, after 24 h of exposure in the BJ cell line, significantly reduced resazurin reduction only at a concentration of 1.000 mg/mL by 52.4% compared with the control (Figure 3B). In the CACO-2 cell line, treatment with the Violeta cultivar extract did not result in any changes in resazurin reduction (Figure 3G). In the SCC-15 cell line, the Violeta extract reduced resazurin reduction at concentrations of 0.500 and 1.000 mg/mL by 41.6% and 75.3%, respectively, compared with the control (Figure 3K).

The Morado garlic cultivar significantly decreased resazurin reduction in the BJ cell line at concentrations of 0.250, 0.500, and 1.000 mg/mL by 45.3%, 99.1%, and 99.0%, respectively, compared with the control (Figure 3D). In the CACO-2 cell line, the Morado cultivar extract decreased resazurin reduction only at the highest concentration (1.000 mg/mL) by 55.4% compared with the control (Figure 3H). In the SCC-15 cell line, all tested concentrations (0.062, 0.125, 0.250, 0.500, and 1.000 mg/mL) of the extract decreased resazurin reduction by 19.8%, 27.2%, 56.6%, 72.9%, and 72.0%, respectively, compared with the control (Figure 3L).

### 2.4. Caspase-3 Activity

**Figure 4:**
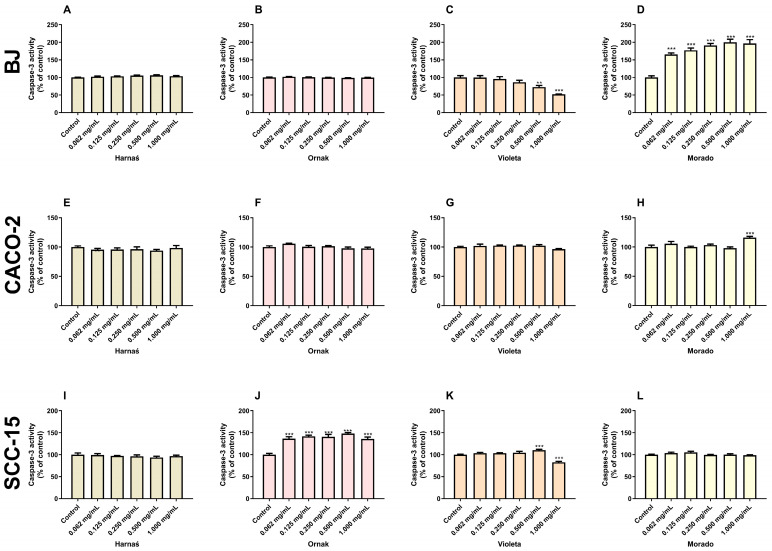
Effect of increasing concentrations (0.062, 0.125, 0.250, 0.500, and 1.000 mg/mL) of extracts from garlic cultivars Harnaś, Ornak, Violeta, and Morado on caspase-3 activity in (**A**–**D**) BJ, (**E**–**H**) CACO-2, and (**I**–**L**) SCC-15 cells after 24 h of exposure. Results are presented as mean ± SD. Statistically significant differences from the control are marked as ** *p* < 0.01 and *** *p* < 0.001.

The experiments showed that after 24 h of exposure, the Harnaś garlic extract did not affect caspase-3 activity in the BJ cell line (Figure 4A). Similarly, in the CACO-2 and SCC-15 cell lines, the Harnaś garlic extract did not alter caspase-3 activity (Figure 4E,I).

The Ornak garlic extract did not affect caspase-3 activity in the BJ and CACO-2 cell lines (Figure 4B,F). However, the extract significantly increased caspase-3 activity at all tested concentrations (0.062, 0.125, 0.250, 0.500, and 1.000 mg/mL) in the SCC-15 cell line, with increases of 36.6%, 41.7%, 40.6%, 48.1%, and 35.9%, respectively, compared with the control (Figure 4J).

Our experiments showed that the Violeta garlic extract decreased caspase-3 activity at concentrations of 0.500 and 1.000 mg/mL by 27.5% and 47.9%, respectively, compared with the control (Figure 4C). In the CACO-2 cell line, the Violeta garlic extract did not cause changes in caspase-3 activity (Figure 4G). Interestingly, in the SCC-15 cell line, a concentration of 0.500 mg/mL increased caspase-3 activity by 10.4% compared with the control, whereas a concentration of 1.000 mg/mL reduced caspase-3 activity by 17.2% (Figure 4K).

The Morado garlic extract increased caspase-3 activity in the BJ cell line at all tested concentrations (0.062, 0.125, 0.250, 0.500, and 1.000 mg/mL) by 65.5%, 77.4%, 90.9%, 100.0%, and 96.7%, respectively, compared with the control (Figure 4D). In the CACO-2 cell line, the Morado garlic extract increased caspase-3 activity only at a concentration of 1.000 mg/mL, with a rise of 16.0% compared with the control (Figure 4H). The tested extract did not affect caspase-3 activity in the SCC-15 cell line (Figure 4L).

### 2.5. Gene Expression

#### 2.5.1. PPARγ and LC3A mRNA Expression in BJ, CACO-2, and SCC-15 Cell Lines

**Figure 5:**
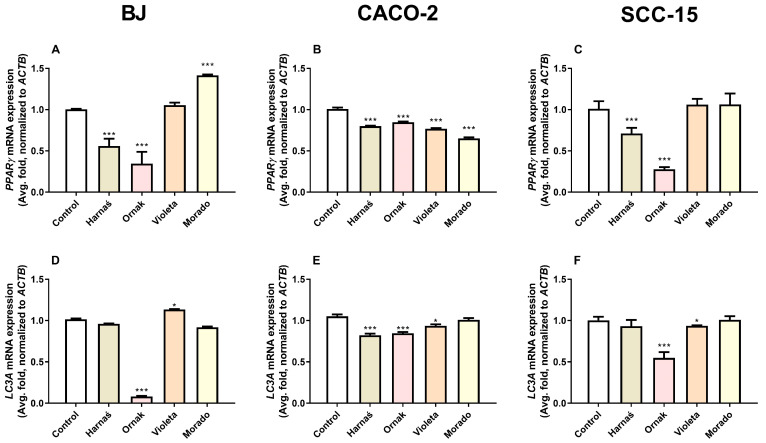
Effect of 0.250 mg/mL of extracts from garlic cultivars Harnaś, Ornak, Violeta, and Morado on mRNA expression of (**A**–**C**) *PPARγ* and (**D**–**F**) *LC3A* in (**A**,**D**) BJ, (**B**,**E**) CACO-2, and (**C**,**F**) SCC-15 cells after 6 h of exposure. *ACTB* was used as the reference gene. Results are presented as mean ± SD. Statistically significant differences from the control are marked as * *p* < 0.05 and *** *p* < 0.001.

Our experiments showed that after 6 h of exposure of BJ cells to 0.250 mg/mL of garlic extracts, the Harnaś and Ornak cultivars significantly decreased *PPARγ* mRNA expression by 44.1% and 65.4%, respectively, compared with the control. Interestingly, the Morado garlic extract significantly increased *PPARγ* mRNA expression by 41.5% compared with the control group (Figure 5A). In the CACO-2 cell line, all tested extracts reduced *PPARγ* mRNA expression: Harnaś by 20.0%, Ornak by 15.3%, Violeta by 23.2%, and Morado by 34.8%, compared with the control (Figure 5B). In the SCC-15 cell line, the Harnaś and Ornak cultivars significantly reduced *PPARγ* mRNA expression by 28.9% and 72.3%, respectively, compared with the control (Figure 5C).

The experiments also showed that after 6 h of exposure of BJ cells to 0.250 mg/mL of the Ornak garlic extract, *LC3A* mRNA expression was significantly decreased by 91.6% compared with the control. However, the Violeta cultivar extract slightly but statistically significantly increased *LC3A* mRNA expression by 13.5% compared with the control group (Figure 5D). In the CACO-2 cell line, exposure to 0.250 mg/mL of Harnaś, Ornak, and Violeta garlic extracts resulted in reduced *LC3A* mRNA expression, with decreases of 17.8%, 15.4%, and 6.7%, respectively, compared with the control (Figure 5E). In the SCC-15 cell line, Ornak and Violeta garlic extracts decreased *LC3A* mRNA expression by 45.3% and 6.4%, respectively, compared with the control (Figure 5F).

#### 2.5.2. CAT and SOD1 mRNA Expression in BJ, CACO-2, and SCC-15 Cell Lines

**Figure 6:**
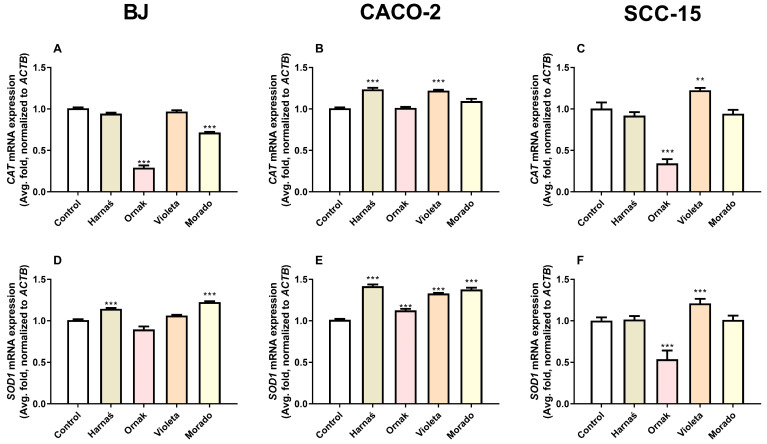
Effect of 0.250 mg/mL of extracts from garlic cultivars Harnaś, Ornak, Violeta, and Morado on mRNA expression of (**A**–**C**) *CAT* and (**D**–**F**) *SOD1* in (**A**,**D**) BJ, (**B**,**E**) CACO-2, and (**C**,**F**) SCC-15 cells after 6 h of exposure. *ACTB* was used as the reference gene. Results are presented as mean ± SD. Statistically significant differences from the control are marked as ** *p* < 0.01 and *** *p* < 0.001.

Our experiments showed that after 6 h of exposure of BJ cells to 0.250 mg/mL of the tested extracts, the Ornak and Morado cultivars significantly decreased *CAT* mRNA expression by 70.8% and 28.4%, respectively, compared with the control (Figure 6A). In the CACO-2 cell line at the same concentration (0.250 mg/mL), extracts from the Harnaś and Violeta cultivars significantly increased *CAT* mRNA expression by 23.8% and 22.2%, respectively, compared with the control (Figure 6B). Interestingly, in the SCC-15 cell line, the Ornak cultivar extract significantly decreased *CAT* mRNA expression by 65.7%, whereas the Violeta cultivar extract increased *CAT* mRNA expression by 22.57% compared with the control (Figure 6C).

For *SOD1* mRNA expression in the BJ cell line, exposure to 0.250 mg/mL of the tested extracts showed that only the Harnaś and Morado cultivars significantly increased *SOD1* mRNA expression, by 14.6% and 22.6%, respectively, compared with the control (Figure 6D). In the CACO-2 cell line, all tested extracts at 0.250 mg/mL increased *SOD1* mRNA expression, with increases of 41.7% (Harnaś), 12.5% (Ornak), 32.9% (Violeta), and 37.8% (Morado), compared with the control (Figure 6E). In the SCC-15 cell line, the Ornak garlic extract significantly decreased *SOD1* mRNA expression by 46.3% compared with the control, while the Violeta garlic extract increased *SOD1* mRNA expression by 21.1% compared with the control (Figure 6F).

### 2.6. Protein Expression

#### 2.6.1. PPARγ and LC3A Protein Expression in BJ, CACO-2, and SCC-15 Cell Lines

**Figure 7:**
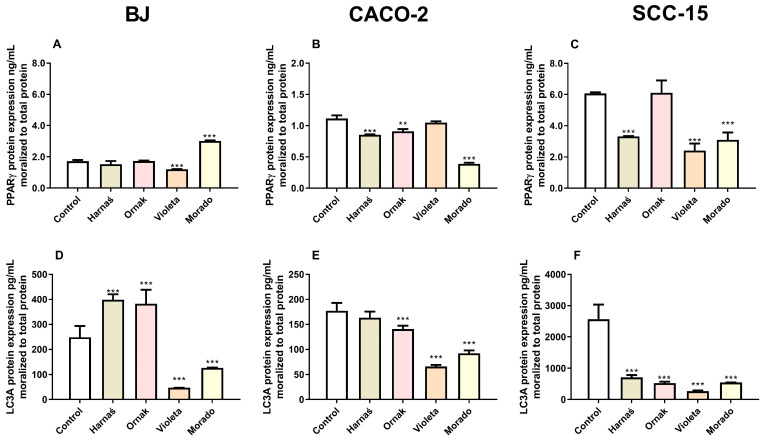
Effect of 0.250 mg/mL of extracts from garlic cultivars Harnaś, Ornak, Violeta, and Morado on protein expression of (**A**–**C**) PPARγ and (**D**–**F**) LC3A in (**A**,**D**) BJ, (**B**,**E**) CACO-2, and (**C**,**F**) SCC-15 cells after 6 h of exposure. Protein levels were normalised to total protein. Results are presented as mean ± SD. Statistically significant differences from the control are marked as ** *p* < 0.01 and *** *p* < 0.001.

Our experiments showed that extracts from the Harnaś and Ornak garlic cultivars significantly increased LC3A protein expression in BJ cells by 150.5 pg/mL and 134.2 pg/mL, respectively, compared with the control. Conversely, extracts from the Violeta and Morado cultivars significantly decreased LC3A protein expression in BJ cells by 201.3 pg/mL and 122 pg/mL, respectively, compared with the control (Figure 7A). In the CACO-2 cell line, extracts from the Ornak, Violeta, and Morado cultivars reduced LC3A protein expression by 36.63 pg/mL, 111.2 pg/mL, and 85.4 pg/mL, respectively, compared with the control (Figure 7B). In the SCC-15 cell line, all tested garlic varieties strongly reduced LC3A protein expression: by 1856.0 pg/mL (Harnaś), 2039.5 pg/mL (Ornak), 2298.0 pg/mL (Violeta), and 2019.2 pg/mL (Morado), compared with the control (Figure 7C).

After 24 h of exposure to the tested extracts in the BJ cell line, only the Violeta cultivar reduced PPARγ protein expression by 0.52 ng/mL compared with the control. Conversely, the Morado cultivar increased PPARγ protein expression by 1.3 ng/mL compared with the control (Figure 7D). In the CACO-2 cell line, a reduction in PPARγ protein expression was observed in the Harnaś, Ornak, and Morado groups, with decreases of 0.3 ng/mL, 0.2 ng/mL, and 0.7 ng/mL, respectively, compared with the control (Figure 7E). In the SCC-15 cell line, PPARγ protein expression decreased in groups treated with Harnaś, Violeta, and Morado garlic extracts, with reductions of 2.8 ng/mL, 3.7 ng/mL, and 3.0 ng/mL, respectively, compared with the control (Figure 7F).

#### 2.6.2. CAT and SOD1 Protein Expression in BJ, CACO-2, and SCC-15 Cell Lines

**Figure 8:**
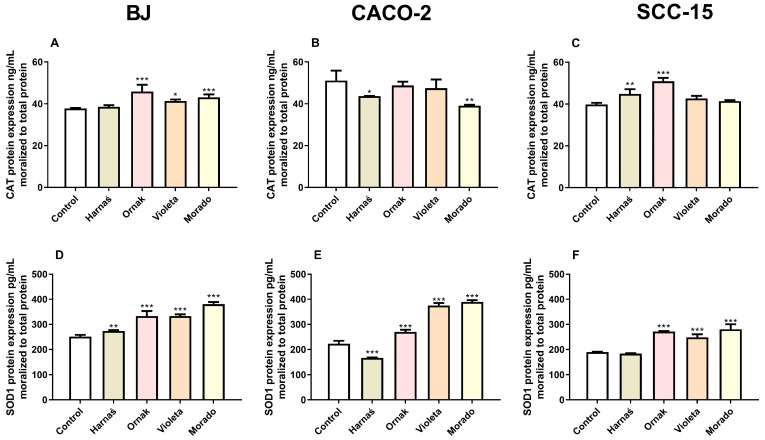
Effect of 0.250 mg/mL of extracts from garlic cultivars Harnaś, Ornak, Violeta, and Morado on protein expression of (**A**–**C**) CAT and (**D**–**F**) SOD1 in (**A**,**D**) BJ, (**B**,**E**) CACO-2, and (**C**,**F**) SCC-15 cells after 6 h of exposure. Protein levels were normalised to total protein. Results are presented as mean ± SD. Statistically significant differences from the control are marked as * *p* < 0.05, ** *p* < 0.01, and *** *p* < 0.001.

Our experiments showed that after 24 h of exposure of BJ cells to the tested extracts, the Ornak, Violeta, and Morado cultivars significantly increased CAT protein expression by 8.1 ng/mL, 3.6 ng/mL, and 5.3 ng/mL, respectively, compared with the control (Figure 8A). In the CACO-2 cell line, extracts from the Harnaś and Morado cultivars decreased CAT protein expression by 7.5 ng/mL and 12.1 ng/mL, respectively, compared with the control (Figure 8B). In the SCC-15 cell line, extracts from the Harnaś and Ornak cultivars increased CAT protein expression by 5.4 ng/mL and 11.1 ng/mL, respectively, compared with the control (Figure 8C).

Our experiments also showed that all tested garlic extracts (Harnaś, Ornak, Violeta, and Morado) significantly increased SOD1 protein expression in the BJ cell line after 24 h of exposure, with increases of 22.4 pg/mL, 81.4 pg/mL, 81.5 pg/mL, and 128.9 pg/mL, respectively, compared with the control (Figure 8D). In the CACO-2 cell line, the Harnaś garlic extract decreased SOD1 protein expression by 56.06 pg/mL compared with the control (Figure 8E). The remaining garlic extracts increased SOD1 protein expression, with increases of 47.6 pg/mL (Ornak), 152.8 pg/mL (Violeta), and 167.3 pg/mL (Morado) compared with the control (Figure 8E). Similarly, in the SCC-15 cell line, the Ornak, Violeta, and Morado garlic extracts increased SOD1 protein expression by 82.4 pg/mL, 58.6 pg/mL, and 91.1 pg/mL, respectively, compared with the control (Figure 8F).

### 2.7. Chromatographic Analysis of Studied Garlic Extracts

The results of the chromatographic analysis of the polyphenolic compounds are summarised in Table 1. The analysis revealed that the Harnaś cultivar is rich in apigenin, catechin, ferulic acid, isoharmentin, sinapic acid, and vanillic acid. The Ornak cultivar was rich in 4-hydroxybenzoic acid. The Violeta cultivar contained high levels of acacetin, epicatechin, hesperidin, hispidulin, myricetin, and naringenin. Finally, the Morado cultivar was the richest in kaempferol, luteolin, and rutin. In the studied extracts, quercetin and rosmarinic acid were not detected (Table 1). Statistical analysis revealed that the garlic cultivar extracts did not differ significantly in terms of the levels of 4-hydroxybenzoic acid, apigenin, epicatechin, hispidulin, kaempferol, luteolin, and naringenin. Among the cultivars, the Violeta cultivar exhibited the most distinct profile compared with the other tested cultivars.

## 3. Discussion

It is well documented that high levels of ROS often cause toxicity or initiate apoptosis [23,24]. Therefore, in the first part of this study, we examined the influence of extracts from the Harnaś, Ornak, Violeta, and Morado garlic cultivars on ROS production in the SCC-15, CACO-2, and BJ cell lines. In our experiments, the Ornak cultivar extract most strongly enhanced ROS production across all studied cell lines. Conversely, the Violeta cultivar induced the smallest increase in ROS production compared with the other tested garlic cultivars. It should also be noted that all tested garlic extracts minimally increased ROS production in the normal BJ cell line compared with the cancerous SCC-15 and CACO-2 cell lines. Previous studies have shown that aged garlic extract increased ROS production in human neuroblastoma SH-SY5Y cells, but when co-treated with 6-hydroxydopamine (6-OHDA), ROS production was reduced [13]. However, it is important to note that while SH-SY5Y is a neuroblastoma cell line, it retains many normal cellular mechanisms and is often considered a model for normal cells [25]. Elevated ROS levels in cancer cells have been shown to initiate cell cycle arrest [26]. Moreover, increased ROS levels contribute to destabilisation of the cancer cell genome, leading to DNA damage and ultimately cell death via necrosis or apoptosis [27]. Ethanolic garlic extracts have been reported to induce ROS-dependent cell death in human triple-negative breast cancer cell lines (MCF-7, MDA-MB-231) without harming normal Vero cells [28]. These extracts were shown to initiate cell cycle arrest and activate apoptosis via caspase-3 involvement [28]. Similarly, Özkan et al. described that small extracellular vesicles isolated from garlic induced ROS-dependent cytotoxicity and apoptosis in human kidney carcinoma (A498) and lung carcinoma (A549) cell lines, while toxicity and apoptosis were significantly lower in normal human dermal fibroblast cell lines [29]. Our previous study demonstrated that extracts from fresh garlic cloves of the Harnaś and Morado cultivars induced ROS-dependent cytotoxicity in SCC-15 cells after 6, 24, and 48 h of exposure, without significant activation of caspase-3 [14]. Our present study also suggests that the tested garlic extracts may contribute to cancer cell death by a ROS-dependent mechanism but activate caspase-3. Differences were likely due to the distinct nature of the extracts used in the previous study.

Due to the presence of ROS in cancer cells often resulting in toxicity, the next stage of the research involved determining cytotoxicity and the impact on cell metabolic status. LDH release from cells into the culture medium was used as an indicator of cytotoxicity. As a cytosolic enzyme, LDH’s presence in the culture medium effectively indicates cell membrane damage or cell death [30]. Conversely, resazurin is a water-soluble dye widely used in in vitro cell tests. This method detects the metabolic activity of cells and can indirectly suggest cell numbers, although this requires further confirmation. Moreover, resazurin is considered a more sensitive alternative to the 3-(4,5-dimethylthiazol-2-yl)-2,5-diphenyltetrazolium bromide (MTT) assay [31]. In our experiments, the Harnaś, Ornak, and Violeta cultivars increased LDH release only at the highest extract concentration (1.000 mg/mL) in the normal BJ cell line. The extract from the Morado cultivar increased LDH release at concentrations of 0.250, 0.500, and 1.000 mg/mL, suggesting that this extract has the strongest toxicity in normal cells. In the SCC-15 cell line, only the Violeta cultivar did not cause LDH release into the culture medium. Interestingly, in the CACO-2 cell line, the Ornak cultivar decreased LDH release across a wide range of concentrations. According to the literature, a decrease in LDH release can occur in vitro with highly toxic compounds that cause rapid cell death, resulting in proteolytic degradation of LDH in the culture medium [32]. Aged garlic extract has been shown to reduce LDH release in normal bovine pulmonary artery endothelial cells, murine macrophage cells (J774), and human umbilical vein endothelial cells when initiated by various stimuli [33,34]. However, at very high concentrations, the extract alone can cause LDH release in normal cells. Conversely, in cancerous cells, garlic extract increases LDH release across a wide range of concentrations. This finding was confirmed in our previous study, which showed increased LDH release in the SCC-15 cell line at concentrations of 0.250 to 1.000 mg/mL, and in another study that showed a similar effect in the MCF-7 and MDA-MB-231 cell lines at concentrations of 0.312 to 1.000 mg/mL [14,35]. Moreover, Isbilen and Volkan reported that at the highest extract concentration, LDH release significantly decreased to levels comparable with the control [35]. Therefore, we can assume that the present data align with the current state of knowledge. In our experiments, the strongest decrease in cell metabolism, as measured by the resazurin reduction test, was observed in BJ and SCC-15 cell lines following treatment with the Ornak and Morado cultivars. In the CACO-2 cell line, the Ornak cultivar increased cell metabolism across a wide range of concentrations, as measured by the resazurin reduction test. Among all the studied cell lines, the Violeta cultivar had the least effect on cell metabolism. Our previous study demonstrated that the Harnaś and Morado cultivars, at concentrations ranging from 0.250 to 1.000 mg/mL, decreased cell numbers in SCC-15 cells, as measured by the Neutral Red assay [14]. Similarly, Siegers et al. [36] reported that garlic extracts inhibited cancer cell proliferation, as measured by the same test, at a concentration of 0.330 mg/mL in the human hepatoma cell line (HepG2) and 0.480 mg/mL in the CACO-2 cell line. A decrease in cell viability, measured using the 3-(4,5-dimethylthiazol-2-yl)-5-(3-carboxymethoxyphenyl)-2-(4-sulfophenyl)-2H-tetrazolium (MTS) assay, was also observed in the A498 and A549 cell lines after treatment with small extracellular vesicles derived from garlic at concentrations ranging from 5 to 50 µg/mL [29]. Additionally, decreases in cell viability and cell numbers were reported in MCF-7 and MDA-MB-231 cells at concentrations ranging from 0.625 to 1.000 mg/mL [35].

In addition to toxicity, apoptosis is another type of cell death, with caspase-3 activity serving as its main marker. Our research shows that the studied garlic extracts had a limited effect on caspase-3 activity. In the BJ cell line, the Morado cultivar increased caspase-3 activity at all studied concentrations (0.062 to 1.000 mg/mL), while the Violeta cultivar slightly decreased caspase-3 activity at the two highest concentrations. In the CACO-2 cell line, almost no changes in caspase-3 activity were observed. In the SCC-15 cell line, no changes in caspase-3 activity were observed with the Harnaś or Morado extracts, which is consistent with our previous studies [14]. Interestingly, the Ornak cultivar increased caspase-3 activity at all studied concentrations. To date, studies conducted by other teams have been consistent in showing that garlic extracts initiate ROS-dependent cell death, although the specific type of cell death (necrosis or apoptosis) depends on factors such as extract concentration, type of extract, or garlic cultivar [29,33,34]. Previous research has shown that aged black garlic extract induces apoptosis in the human gastric cancer cell line (SGC-7901) at a concentration of 100 mg/mL [37]. Similarly, Özkan et al. reported that garlic-derived small extracellular vesicles at a concentration of 50 µg/mL increased *Caspase-3*, *Caspase-9*, and *P53* mRNA levels in A498 and A549 cell lines, confirming ROS-dependent apoptosis [29]. Apoptosis, measured by caspase-3 and caspase-9 activity, has also been confirmed in MCF-7 and MDA-MB-231 cell lines at concentrations ranging from 0.156 to 1.000 mg/mL [28,35].

It is well established that garlic is rich in various polyphenols, which contribute to its numerous health-promoting properties [38]. In addition, some studies have attempted to correlate the polyphenol content with antiproliferative properties [39]. However, it should be noted that these results are inconclusive because the authors tested entirely different garlic varieties without conducting such detailed chromatographic analyses. Our previous study linked the strong cytotoxic properties of the Morado cultivar to its suspected high polyphenol content. Several polyphenols have been described as PPARγ agonists. Particularly, (+)-catechin [40], or kaempferol, are well-known full and partial PPARγ agonists, respectively [41]. Interestingly, the current study revealed that the Morado cultivar did not contain catechins. However, some studies have shown that aged garlic extract initiates various cellular processes through PPARγ pathways [20,21,22]. Therefore, based on previously described findings, we selected PPARγ and enzymes related to the PPARγ pathway, such as LC3A, SOD1, and CAT, for further study. Our data showed that in the normal BJ cell line, only the Morado cultivar extract increased both *PPARγ* mRNA and PPARγ protein expression while decreasing LC3A protein expression. Interestingly, the Violeta cultivar did not significantly affect *PPARγ* or *LC3A* mRNA expression but strongly decreased PPARγ and LC3A protein expression. Both the Harnaś and Ornak cultivars acted similarly, significantly decreasing *PPARγ* mRNA expression while increasing LC3A protein expression. In the CACO-2 cell line, extracts from all studied cultivars (Harnaś, Ornak, Violeta, and Morado) similarly decreased both PPARγ and LC3A mRNA and protein expression. Our research shows that the Morado cultivar had the strongest effect on decreasing the expression of both studied proteins. Finally, in the SCC-15 cell line, the Harnaś and Ornak cultivars decreased *PPARγ* mRNA expression, while reductions in PPARγ protein levels were observed with the Harnaś, Violeta, and Morado cultivars. Interestingly, all the studied extracts strongly decreased LC3A protein expression.

Our findings allow us to establish certain relationships. Generally, in the studied cancerous cell lines (CACO-2 and SCC-15), the garlic cultivars decreased both PPARγ and LC3A protein expression. By contrast, in the normal BJ cells, the Harnaś and Ornak extracts did not affect PPARγ protein expression but increased LC3A protein expression. The Violeta extract acted similarly to its effects in cancerous cell lines, while the Morado extract increased PPARγ protein expression but decreased LC3A protein expression. Our chromatographic analysis revealed that the Harnaś and Ornak extracts had similar polyphenolic compositions, whereas the Violeta and Morado extracts were quite distinct. We believe this may explain the differences observed in normal cells. Moreover, the highest levels of catechin, a known PPARγ agonist, were detected in the Harnaś and Ornak cultivars. Therefore, we propose that the observed changes in protein expression and toxicity, at least in the case of the Harnaś and Ornak cultivars, are likely due to PPARγ activation. Moreover, our analysis revealed that the studied extracts differed significantly in their polyphenolic compound content. Large differences in polyphenolic levels have previously been reported in endemic Italian garlic cultivars (Schiacciato, Bianco, Torella, Salomone, and Ufita), even when grown on the same test field [12]. Similarly, substantial variations in polyphenolic content were observed in 43 Chinese garlic cultivars [42]. To date, it has been described that *p*-coumaric acid, ellagic acid, ferulic acid, gallic acid, apigenin, and vanillic acid strongly decrease *Pparγ* mRNA expression in 3T3-L1 mouse preadipocytes during differentiation [43]. By contrast, hesperidin, quercetin, resveratrol, and curcumin were shown to slightly decrease *Pparγ* mRNA expression, although the effects were not statistically significant. These findings support our hypothesis.

Our study is the first that tried to correlate the polyphenolic content of garlic with changes in PPARγ protein expression. Previously, aged black garlic extract was reported to decrease *Pparγ* mRNA expression in the adipose tissue of rats [28]. Unfortunately, most research focuses on sulfur compounds derived from garlic. For instance, alliin has been shown to ameliorate gut inflammation through PPARγ pathway activation in the RAW 264.7 mouse cell line [44]. Garlic-derived diallyl disulfide has been demonstrated to increase peroxisome proliferator-activated receptor gamma coactivator 1-alpha (PGC1α) expression in a ROS-dependent manner, inducing mitochondrial biogenesis at an early stage of treatment that precedes cell cycle arrest and apoptosis in the human neuroblastoma (SH-SY5Y) cell line [22]. The increase in PGC1α expression also suggests PPARγ activation. Therefore, we propose that both polyphenolic and organosulfur compounds contribute to PPARγ activation and the modulation of related pathways.

As mentioned above, the mechanism of action of garlic and polyphenols is accompanied by an increase in ROS production. Therefore, in the last part of our work, we examined the mRNA and protein expression of antioxidant enzymes such as SOD1 and CAT, whose expression is controlled by PPARγ. Our experiments showed that the Ornak, Violeta, and Morado cultivars acted similarly in the BJ, CACO-2, and SCC-15 cell lines. These garlic cultivars increased SOD1 protein expression. Interestingly, the pattern of *SOD1* mRNA expression did not fully align with the protein expression. Minor discrepancies between mRNA and protein levels may be caused by differences in the expression intensity of the studied proteins. It is well established that mRNA should be measured earlier than protein [45]. Moreover, high mRNA expression is often accompanied by lower protein levels and vice versa, usually as a result of feedback effects. The Harnaś cultivar appears to act differently, increasing both *SOD1* mRNA and SOD1 protein expression in BJ cells. In the CACO-2 cell line, an increase in *SOD1* mRNA expression was observed, but this was accompanied by a decrease in SOD1 protein expression. Interestingly, in the SCC-15 cell line, no significant changes were observed at either the mRNA or protein level. For CAT protein expression, the Harnaś, Ornak, Violeta, and Morado cultivars either increased or had no effect on CAT protein levels in BJ and SCC-15 cell lines. A notable observation was that the strong decrease in *CAT* mRNA expression caused by the Ornak cultivar was accompanied by an increase in CAT protein expression. An opposite phenomenon was observed in the CACO-2 cell line, where the Harnaś and Violeta cultivars increased *CAT* mRNA expression but slightly decreased CAT protein expression. To date, it has been reported that in rats, after 2 weeks on a diet containing 2% garlic, a decrease in *Cat* mRNA and CAT protein expression was observed in the kidney and liver [18]. This diet did not significantly affect SOD activity in kidney or liver tissues [18]. Similarly, a decrease in CAT activity was observed in rat liver after 7 days of treatment with fresh garlic [46]. However, the response strongly depends on the type of tissue. In rats fed fresh garlic homogenate for 30 days, cardioprotective effects were observed [47]. Garlic prevented oxidative stress and mitigated decreases in CAT and SOD activity in the rat heart induced by the cytostatic agent doxorubicin. Similar protective effects of aged garlic extract were observed in the rat liver, where aged garlic extract prevented ethephon-induced decreases in CAT and SOD activity [48]. Discrepancies between studies are likely due to differences in the garlic dose, cultivar, type of cells, and exposure duration. In normal human osteoarthritic chondrocytes, allicin, sulforaphane, and lycopene derived from garlic increased protein expression of SOD1 and CAT [49]. In human breast cancer (MCF-7) cell lines inoculated in mice, black garlic extracts were shown to increase SOD activity in the serum of the mice [37]. Additionally, 120 µM diallyl disulfide has been reported to increase *SOD1* mRNA expression in human colon cancer HT-29 cells, which, according to the authors, results in cell cycle arrest and apoptosis [50]. Therefore, we can conclude that our findings are consistent with the current state of knowledge on the impact of garlic extracts on SOD and CAT expression and activity.

## 4. Materials and Methods

### 4.1. Reagents

Minimum Essential Medium (MEM), Dulbecco’s Modified Eagle Medium/Nutrient Mixture F-12 (DMEM/F12), and phosphate-buffered saline without Ca^2+^ and Mg^2+^ (PBS) were purchased from Corning (Manassas, VA, USA). Real-time polymerase chain reaction (RT-PCR) TaqMan probes corresponding to a specific nucleotide sequence encoding—CAT (Hs00156308_m1), SOD1 (Hs00533490_m1), LC3A (Hs01076567_g1), PPARγ (Hs00234592_m1), and ACTB (Hs01060665_g1)—and a High-Capacity cDNA Reverse Transcription Kit were obtained from Life Technologies (Paisley, UK). Trypsin, penicillin, streptomycin, radioimmunoprecipitation assay buffer (RIPA), staurosporine, and dimethyl sulfoxide were purchased from Sigma-Aldrich (St. Louis, MO, USA). The substrate for measuring caspase-3 activity (235400) was purchased from Merck Millipore (Darmstadt, Germany). The 6-, 12-, and 96-well culture plates were purchased from TPP Techno Plastic Products AG (Trasadingen, Switzerland). The kit for determining lactate dehydrogenase (LDH) release (11644793001) was obtained from Roche Applied Science (Munich, Germany). Basic laboratory reagents, including acids, bases, salts, alcohols, and simple chemical compounds, were purchased from POCH S.A. (Gliwice, Poland). Foetal bovine serum (FBS), a Universal RNA Purification Kit (E3598-02), and quantitative PCR (qPCR) master mix (Fast Probe qPCR Master Mix) were obtained from EURx (Gdańsk, Poland). Enzyme-linked immunosorbent assay (ELISA) kits for PPARγ (H1361), SOD1 (H1113), CAT (H0643), and LC3A were purchased from Elabscience Biotechnology (Wuhan, China).

### 4.2. Preparation of Extracts

The Harnaś, Ornak, Violeta, and Morado garlic cultivars were kindly donated by Krzysztof Markiewicz from the Markie-Pol company (Dąbrówka Wielka, Poland). Two varieties, Harnaś and Ornak, were grown in Poland in the Świętokrzyskie Voivodeship, while Morado and Violeta were cultivated on the Iberian Peninsula in Spain, in the commune of Las Pedroñeras. The garlic was lyophilised, powdered, and stored at −30 °C. Aqueous extracts (in PBS) were prepared from the lyophilised powder according to a previously described procedure [51]. Briefly, 10 g of lyophilised garlic powder was mixed with 100 mL of PBS and blended in an ice bath. The extracts were then incubated for 30 min at 4 °C and centrifuged for 10 min at 3900× *g*. The supernatants were collected and sterilised by passing them through a 0.22-μm filter (Merck Millipore). The extracts were stored in aliquots at −20 °C.

### 4.3. Cell Culture and Experiments

Human squamous cell carcinoma (SCC-15 (CRL-1623)), colon adenocarcinoma (CACO-2 (HTB-37)), and normal skin fibroblast (BJ (CRL-2522)) cell lines were obtained from the American Type Culture Collection (distributor: LGC Standards, Łomianki, Poland). The SCC-15 cell line was maintained in DMEM/F12, while the BJ and CACO-2 cell lines were maintained in MEM. The media used in the experiments were phenol red-free and contained 2.5 mM L-glutamine. Additionally, the media were supplemented with 10% FBS, and for SCC-15 cells, 400 ng/mL hydrocortisone was also included. The cells were maintained at 37 °C in a humidified atmosphere with 5% CO_2_. The cells were seeded into 96-well culture plates (Costar, St. Louis, MO, USA) at a density of 6 × 10^3^ per well (for the 24 h treatment) and initially cultured for 24 h prior to the experiment. Subsequently, the medium was replaced with fresh medium containing increasing concentrations of extracts from garlic cultivars (*Allium sativum* L.)—Harnaś, Ornak, Violeta, and Morado—at 0.062, 0.125, 0.250, 0.500, and 1.000 mg/mL for 24 h. Afterwards, analyses were performed according to the protocols described below.

### 4.4. ROS Measurement

In our study, the fluorogenic dye H_2_DCFDA was used to detect intracellular ROS, following a previously described protocol [14]. Briefly, 5 μM H_2_DCFDA was applied in serum-free medium for 45 min before treatment with the extracts. After 24 h of incubation with the garlic cultivar extracts (0.062, 0.125, 0.250, 0.500, and 1.000 mg/mL), fluorescence was measured using a microplate readerFilterMax F5 Multi Mode (Molecular Devices, Corp., Sunnyvale, CA, USA). Fluorescence was measured with excitation at 485 nm and emission at 535 nm.

### 4.5. LDH Release

The level of LDH release was measured on a 96-well plate after 24 h of incubation with garlic cultivar extracts (0.062, 0.125, 0.250, 0.500, and 1.000 mg/mL). The toxicity of the extracts was assessed following a previously described protocol [52]. Briefly, after exposing the cells to the extracts, the culture medium was transferred to a new 96-well plate. The plate containing the cells was frozen at −80 °C for subsequent caspase-3 activity analysis. A reaction solution was added to the medium on the new plate and incubated for 30 min at room temperature in the dark. The colorimetric product was then measured. Absorbance measurements were taken at 490 nm.

### 4.6. Resazurin Reduction Assay

The resazurin measurement was conducted according to a previously described protocol [53]. Briefly, the cells were incubated with increasing concentrations of garlic cultivar extracts for 6, 24, or 48 h. The medium was then replaced with fresh medium containing 10% resazurin and 1% FBS. After 60 min of incubation, fluorescence was measured. Fluorescence was measured with excitation at 530 nm and emission at 590 nm.

### 4.7. Caspase-3 Activity Assay

The caspase-3 activity was measured according to a previously described protocol [54] with minor modifications. Briefly, after the LDH release experiments, the 24 h medium was removed and the cells were frozen at −80 °C until measurement. After thawing, the cells were lysed using lysis buffer (50 mM HEPES, pH 7.4, 100 mM NaCl, 0.1% CHAPS, 1 mM EDTA, 10% glycerol, and 10 mM DTT) at 10 °C for 10 min. The lysates were then incubated with the caspase-3 substrate Ac-DEVD-pNA in the same buffer at 37 °C. After 30 min, absorbance was measured. Absorbance measurements were taken at 405 nm.

### 4.8. Gene Expression Analysis by RT-PCR

Cells (BJ, CACO-2, or SCC-15) were treated with 0.250 mg/mL of extracts from the Harnaś, Ornak, Violeta, and Morado garlic cultivars for 6 h. Total RNA was then extracted using the Universal RNA Purification Kit, following the manufacturer’s protocol. RNA quality and quantity were assessed using a Nanodrop device. The reverse transcription reaction was performed according to the manufacturer’s instructions (ThermoFisher, Waltham, MA, USA) in a final volume of 20 μL, using 800 ng of RNA as the cDNA template. RT-PCR was conducted using the Fast Probe qPCR Master Mix (2x) kit (EURx, Gdańsk, Poland) with TaqMan probes specific for the genes PPARγ, SOD1, CAT, LC3A, and ACTB in a total volume of 20 μL, including 1 μL of cDNA. The standard qPCR procedure was as follows: 3 min at 95 °C, followed by 40 cycles of 10 s at 95 °C and 30 s at 55 °C. ACTB was used as the reference gene, and the RefFinder (http://blooge.cn/RefFinder/, access date: 14 April 2023) web-based tool was employed to evaluate reference gene expression.

### 4.9. ELISA

The levels of PPARγ, SOD1, CAT, and LC3A proteins were determined after 24 h of treatment with 0.250 mg/mL of extracts from the Harnaś, Ornak, Violeta, and Morado garlic cultivars via ELISA. The expression of the studied proteins was determined in cell culture lysates collected in RIPA. ELISA was performed according to the manufacturer’s instructions. Briefly, a 96-well plate was pre-coated with monoclonal antibodies specific to PPARγ, SOD1, CAT, and LC3A proteins. Standards and collected cell extracts were incubated for 90 min at 37 °C in the pre-coated 96-well plate. After removing the liquid, 100 µL of biotinylated detection antibodies was added and incubated for 60 min. The plate was washed three times to remove unbound substances, and horseradish peroxidase-conjugated avidin was added. Following additional washing, in the final step, 90 µL of substrate solution was added to the wells and incubated for 15 min. Then, 50 µL of stop solution was added, and the absorbance was measured. The absorbance value was proportional to the levels of the studied proteins. Protein levels were measured and standardised using a Nanodrop device. Absorbance measurements were taken at 450 nm.

### 4.10. Chromatographic Determination of Biologically Active Compounds

Analysis of polyphenolic compounds in garlic extracts was performed using a Prominence-i LC 2030C D3 Plus system (Shimadzu, Kyoto, Japan) with a diode array detector and a Luna Omega 5-μm Polar C18, 100 Å, 250 × 10 mm Phenomenex column (Torrance, CA, USA). The mobile phase flow rate was 1.2 mL/min, and the sample injection volume was 20 μL. The mobile phase consisted of two mixtures: mixture A (distilled water acidified with 0.1% formic acid) and mixture B (methanol acidified with 0.1% formic acid). Each analysis lasted 60 min and followed these gradient conditions: 20% to 40% B over 10 min, 40% B for 10 min, 40% to 50% B over 10 min, 50% to 60% B over 5 min, 60% B for 5 min, 60% to 70% B over 5 min, 70% to 90% B over 5 min, 90% B for 5 min, 90% to 20% B (initial condition) over 1 min, and 20% B for 4 min. The process was carried out at 40 °C. Polyphenolic compounds were identified at the following wavelengths: 254 nm (myricetin, quercetin, luteolin, isoharmentil), 256 nm (rutin), 260 nm (vanillalic acid), 264 nm (kaempferol) 267 nm (apigenin, acacetin) 271 nm (gallic acid), 274 nm (syringic acid), 278 nm (catechin, epicatechin), 283 nm (naringenin), 284 nm (hesperidin), 310 nm (*p*-coumaric acid), 323 nm (caffeic acid, ferulic acid, sinapinic acid), 326 nm (chlorogenic acid), and 329 nm (rosmarinic acid). Representative chromatograms have been added as a Supplementary File. A calibration curve of standards was prepared to quantify the tested compounds. Standard solutions were prepared in 70% methanol acidified with 0.1% formic acid (POCh, Katowice, Poland) at a concentration of 100 µg/mL. Stock standard solutions were diluted to obtain final concentrations in the range of 0.5 to 4.0 µg/mL. All solutions were filtered through a membrane filter (PTFE 25 mm, 0.22 μm; ChemLand, Starogard, Poland) [55]. Selected polyphenolic compounds were interpreted using LabSolution ver. 5.93 software (Shimadzu Corporation, Kyoto, Japan).

### 4.11. Statistical Analysis

The data were normalised to vehicle-treated controls (% of control). Results are presented as mean ± standard deviation (SD) from three independent experiments, with each treatment repeated at least three times. Statistical analysis was performed using GraphPad Prism 8 software with a one-way analysis of variance (ANOVA) with Tukey’s multiple comparison post hoc test. Statistically significant differences between individual values were denoted as follows: * *p* < 0.05, ** *p* < 0.01, *** *p* < 0.001. In the case of chromatographical one-way analysis of variance (ANOVA) with Dunnett’s intergroup post hoc test, the same letters (a, b, or c) indicate groups that do not differ statistically significantly at *p* < 0.05 in the analysis.

## 5. Conclusions

**Figure 9:**
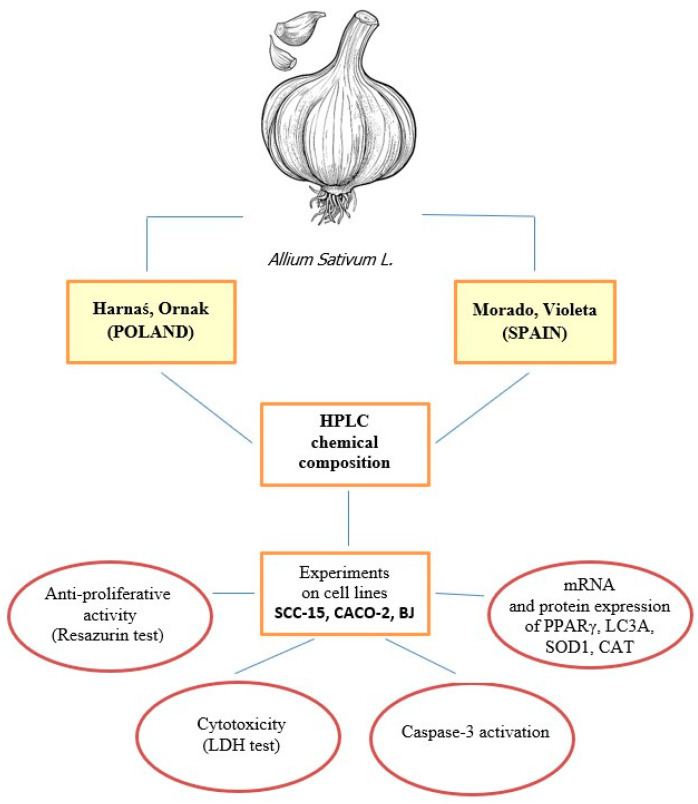
Proposed mechanism of action of the tested garlic extracts.

Our study demonstrated that high ROS production was correlated with the strong toxicity of the studied garlic extracts. The conducted experiments showed that all the studied extracts produced a lesser increase in ROS in normal BJ fibroblasts and were less toxic to these cells. The expression patterns of PPARγ, LC3A, SOD1, and CAT, as well as the chromatographic findings, suggest different mechanisms of action for the studied garlic cultivars (Figure 9). The current study showed the health-promoting and anticancer properties of garlic. The highest levels of catechin, a known PPARγ agonist, were detected in the Harnaś and Ornak cultivars, correlating with changes in PPARγ and LC3A protein expression. Unfortunately, we could not definitively identify which polyphenol, or how it is involved, contributes to PPARγ activation. Therefore, further studies are required to elucidate the role of PPARγ in the mechanism of action of garlic extracts.

## Figures and Tables

**Table 1:** Results of quantitative analysis of polyphenolic compounds (presented as µg/mL± SD) in extracts obtained from garlic cultivars Harnaś, Ornak, Violeta, and Morado. Compounds identified in the extracts are listed. nd indicates compounds that were not detected. The highest values are bolded. The same letters (a, b, or c) indicate groups that do not differ statistically significantly at *p* < 0.05. If necessary, the conversion to mg/kg is given by the formula: μg/mL × (volume of solution in L)/(mass of sample in g) = mg/kg. Volume of solution = 100 mL, mass of sample = 10 g.

Compounds	Harnaś [µg/mL]	Ornak [µg/mL]	Violeta [µg/mL]	Morado [µg/mL]
4-hydroxybenzoic acid	0.181 ± 0.107 ^a^	**0.399 ± 0.208 ^a^**	0.185 ± 0.001 ^a^	0.347 ± 0.245 ^a^
Acacetin	0.091 ± 0.001 ^a^	0.308 ± 0.036 ^b^	**0.863 ± 0.001 ^c^**	0.209 ± 0.103 ^ab^
Apigenin	**0.429 ± 0.001 ^a^**	0.298 ± 0.154 ^a^	0.307 ± 0.173 ^a^	0.238 ± 0.069 ^a^
Caffeic acid	nd	0.052 ± 0.037 ^a^	nd	0.052 ± 0.037 ^a^
Catechin	**3.892 ± 0.016 ^a^**	3.189 ± 1.554 ^ab^	0.553 ± 0.006 ^b^	nd
Chrogenic acid	nd	0.067 ± 0.047 ^a^	nd	0.067 ± 0.047 ^a^
Epicatechin	1.460 ± 0.013 ^a^	1.171 ± 0.828 ^a^	**1.775 ± 0.011 ^a^**	1.171 ± 0.828 ^a^
Ferulic acid	**0.055 ± 0.001 ^a^**	nd	nd	nd
Gallic acid	nd	nd	nd	3.236 ± 2.288 ^a^
Hesperidin	0.100 ± 0.000 ^a^	nd	**0.065 ± 0.045 ^a^**	nd
Hispidulin	0.418 ± 0.007 ^a^	0.393 ± 0.005 ^a^	**0.426 ± 0.001 ^a^**	0.282 ± 0.161 ^a^
Isoharmentin	**0.200 ± 0.004 ^a^**	0.012 ± 0.016 ^b^	nd	0.129 ± 0.074 ^ab^
Kaempferol	0.233 ± 0.001 ^a^	0.249 ± 0.024 ^a^	0.245 ± 0.003 ^a^	**0.329 ± 0.089 ^a^**
Luteolin	0.260 ± 0.001 ^a^	0.259 ± 0.083 ^a^	0.348 ± 0.007 ^a^	**0.550 ± 0.494 ^a^**
Myricetin	0.615 ± 0.001 ^a^	0.569 ± 0.402 ^a^	**1.796 ± 0.086 ^b^**	0.569 ± 0.402 ^a^
Naringenin	0.678 ± 0.002 ^a^	1.349 ± 0.849 ^a^	**2.590 ± 0.005 ^a^**	1.275 ± 0.901 ^a^
p-Coumaric acid	0.045 ± 0.000 ^a^	nd	nd	nd
Quercetin	nd	nd	nd	nd
Rosmarinic acid	nd	nd	nd	nd
Rutin	0.176 ± 0.007 ^a^	nd	nd	**0.207 ± 0.293 ^a^**
Sinapic acid	**0.085 ± 0.000 ^a^**	nd	0.069 ± 0.001 ^b^	nd
Syrigynic acid	nd	0.064 ± 0.045 ^a^	0.064 ± 0.001 ^a^	0.064 ± 0.045 ^a^
Vanillic acid	**0.059 ± 0.000 ^a^**	0.047 ± 0.033 ^a^	nd	0.047 ± 0.033 ^a^

## Data Availability

Dataset available on request from the authors.

## Supplementary Materials

The following supporting information can be downloaded at https://www.mdpi.com/article/10.3390/ijms26010387/s1.

## References

[1] Skoczylas J., Jędrszczyk E., Dziadek K., Dacewicz E., Kopeć A. (2023). Basic Chemical Composition, Antioxidant Activity and Selected Polyphenolic Compounds Profile in Garlic Leaves and Bulbs Collected at Various Stages of Development. Molecules.

[2] Chidike Ezeorba T.P., Ezugwu A.L., Chukwuma I.F., Anaduaka E.G., Udenigwe C.C. (2024). Health-Promoting Properties of Bioactive Proteins and Peptides of Garlic (*Allium sativum*). Food Chem..

[3] Gambelli L., Marconi S., Durazzo A., Camilli E., Aguzzi A., Gabrielli P., Marletta L., Lisciani S. (2021). Vitamins and Minerals in Four Traditional Garlic Ecotypes (*Allium sativum* L.) from Italy: An Example of Territorial Biodiversity. Sustainability.

[4] Pacholczyk-Sienicka B., Modranka J., Ciepielowski G. (2024). Comparative Analysis of Bioactive Compounds in Garlic Owing to the Cultivar and Origin. Food Chem..

[5] Bhatwalkar S.B., Mondal R., Krishna S.B.N., Adam J.K., Govender P., Anupam R. (2021). Antibacterial Properties of Organosulfur Compounds of Garlic (*Allium sativum*). Front. Microbiol..

[6] Rouf R., Uddin S.J., Sarker D.K., Islam M.T., Ali E.S., Shilpi J.A., Nahar L., Tiralongo E., Sarker S.D. (2020). Antiviral Potential of Garlic (*Allium sativum*) and Its Organosulfur Compounds: A Systematic Update of Pre-Clinical and Clinical Data. Trends Food Sci. Technol..

[7] Khounganian R.M., Alwakeel A., Albadah A., Nakshabandi A., Alharbi S., Almslam A.S. (2023). The Antifungal Efficacy of Pure Garlic, Onion, and Lemon Extracts Against Candida Albicans. Cureus.

[8] Sharma V., Sinha E.S., Singh J. (2024). Investigation of In-Vitro Anti-Cancer and Apoptotic Potential of Garlic-Derived Nanovesicles against Prostate and Cervical Cancer Cell Lines. Asian Pac. J. Cancer Prev..

[9] Pandey P., Khan F., Alshammari N., Saeed A., Aqil F., Saeed M. (2023). Updates on the Anticancer Potential of Garlic Organosulfur Compounds and Their Nanoformulations: Plant Therapeutics in Cancer Management. Front. Pharmacol..

[10] Apel K., Hirt H. (2004). Reactive Oxygen Species: Metabolism, Oxidative Stress, and Signal Transduction. Annu. Rev. Plant Biol..

[11] Perillo B., Di Donato M., Pezone A., Di Zazzo E., Giovannelli P., Galasso G., Castoria G., Migliaccio A. (2020). ROS in Cancer Therapy: The Bright Side of the Moon. Exp. Mol. Med..

[12] Fratianni F., Ombra M.N., Cozzolino A., Riccardi R., Spigno P., Tremonte P., Coppola R., Nazzaro F. (2016). Phenolic Constituents, Antioxidant, Antimicrobial and Anti-Proliferative Activities of Different Endemic Italian Varieties of Garlic (*Allium sativum* L.). J. Funct. Foods.

[13] Kohda K., Goda H., Itoh K., Samejima K., Fukuuchi T. (2013). Aged Garlic Extract Reduces ROS Production and Cell Death Induced by 6-Hydroxydopamine through Activation of the Nrf2-ARE Pathway in SH-SY5Y Cells. Pharmacol. Pharm..

[14] Szychowski K.A., Binduga U.E., Rybczyńska-Tkaczyk K., Leja M.L., Gmiński J. (2018). Cytotoxic Effects of Two Extracts from Garlic (*Allium sativum* L.) Cultivars on the Human Squamous Carcinoma Cell Line SCC-15. Saudi J. Biol. Sci..

[15] Rupérez A.I., Anguita-Ruiz A. (2018). Genetics of Oxidative Stress and Obesity-Related Diseases. Obesity.

[16] Elrod H.A., Sun S.-Y. (2008). PPARγ and Apoptosis in Cancer. PPAR Res..

[17] Guo Y., Zhang K., Wang Q., Li Z., Yin Y., Xu Q., Duan W., Li C. (2011). Neuroprotective Effects of Diallyl Trisulfide in SOD1-G93A Transgenic Mouse Model of Amyotrophic Lateral Sclerosis. Brain Res..

[18] Pedraza-Chaverrí J., Granados-Silvestre M.D., Medina-Campos O.N., Maldonado P.D., Olivares-Corichi I.M., Ibarra-Rubio M.E. (2001). Post-Transcriptional Control of Catalase Expression in Garlic-Treated Rats. Mol. Cell. Biochem..

[19] Chu Y.-L., Ho C.-T., Chung J.-G., Rajasekaran R., Sheen L.-Y. (2012). Allicin Induces P53-Mediated Autophagy in Hep G2 Human Liver Cancer Cells. J. Agric. Food Chem..

[20] Morihara N., Ide N., Weiss N. (2010). Aged Garlic Extract Inhibits CD36 Expression in Human Macrophages via Modulation of the PPARgamma Pathway. Phytother. Res..

[21] de Carvalho M.V., Gonçalves-de-Albuquerque C.F., Silva A.R. (2021). PPAR Gamma: From Definition to Molecular Targets and Therapy of Lung Diseases. Int. J. Mol. Sci..

[22] Pagliei B., Aquilano K., Baldelli S., Ciriolo M.R. (2013). Garlic-Derived Diallyl Disulfide Modulates Peroxisome Proliferator Activated Receptor Gamma Co-Activator 1 Alpha in Neuroblastoma Cells. Biochem. Pharmacol..

[23] Redza-Dutordoir M., Averill-Bates D.A. (2016). Activation of Apoptosis Signalling Pathways by Reactive Oxygen Species. Biochim. Biophys. Acta Mol. Cell Res..

[24] Glorieux C., Liu S., Trachootham D., Huang P. (2024). Targeting ROS in Cancer: Rationale and Strategies. Nat. Rev. Drug Discov..

[25] Kovalevich J., Santerre M., Langford D. (2013). Considerations for the Use of SH-SY5Y Neuroblastoma Cells in Neurobiology. Methods Mol. Biol..

[26] Sahoo B.M., Banik B.K., Borah P., Jain A. (2022). Reactive Oxygen Species (ROS): Key Components in Cancer Therapies. Anticancer. Agents Med. Chem..

[27] Li Z., Yang Y., Ming M., Liu B. (2011). Mitochondrial ROS Generation for Regulation of Autophagic Pathways in Cancer. Biochem. Biophys. Res. Commun..

[28] Upadhyay S., Ahmad R., Kumar R., Ghildiyal S., Singh A., Ahmad K., Husain I., Barkat M.A., Hassan M.Z., Asiri Y.I. (2024). Exploring the ROS-Mediated Anti-Cancer Potential in Human Triple-Negative Breast Cancer by Garlic Bulb Extract: A Source of Therapeutically Active Compounds. J. Tradit. Complement. Med..

[29] Özkan İ., Koçak P., Yıldırım M., Ünsal N., Yılmaz H., Telci D., Şahin F. (2021). Garlic (*Allium sativum*)-Derived SEVs Inhibit Cancer Cell Proliferation and Induce Caspase Mediated Apoptosis. Sci. Rep..

[30] Decker T., Lohmann-Matthes M.-L. (1988). A Quick and Simple Method for the Quantitation of Lactate Dehydrogenase Release in Measurements of Cellular Cytotoxicity and Tumor Necrosis Factor (TNF) Activity. J. Immunol. Methods.

[31] Pereira M.I.A., Monteiro C.A.P., de Oliveira W.F., Santos B.S., Fontes A., Cabral Filho P.E. (2020). Resazurin-Based Assay to Evaluate Cell Viability After Quantum Dot Interaction. Methods Mol. Biol..

[32] Kendig D.M., Tarloff J.B. (2007). Inactivation of Lactate Dehydrogenase by Several Chemicals: Implications for in Vitro Toxicology Studies. Toxicol. Vitr..

[33] Ide N., Lau B.H.S., Ryu K., Matsuura H., Itakura Y. (1999). Antioxidant Effects of Fructosyl Arginine, a Maillard Reaction Product in Aged Garlic Extract. J. Nutr. Biochem..

[34] Ide N., Lau B.H.S. (2001). Garlic Compounds Minimize Intracellular Oxidative Stress and Inhibit Nuclear Factor-ΚB Activation. J. Nutr..

[35] Isbilen O., Volkan E. (2021). Allium Willeanum Holmboe Exerts Anticancer Activities on Metastatic Breast Cancer Cells MCF-7 and MDA-MB-231. Heliyon.

[36] Siegers C.P., Steffen B., Röbke A., Pentz R. (1999). The Effects of Garlic Preparations against Human Tumor Cell Proliferation. Phytomedicine.

[37] Wang X., Jiao F., Wang Q.-W.W., Wang J., Yang K., Hu R.-R.R., Liu H.-C.C., Wang H.-Y.Y., Wang Y.-S.S. (2012). Aged Black Garlic Extract Induces Inhibition of Gastric Cancer Cell Growth in Vitro and in Vivo. Mol. Med. Rep..

[38] Škrovánková S., Mlček J., Snopek L., Planetová T. (2018). Polyphenols and Antioxidant Capacity in Different Types of Garlic. Potravin. Slovak J. Food Sci..

[39] Furdak P., Pieńkowska N., Kapusta I., Bartosz G., Sadowska-Bartosz I. (2023). Comparison of Antioxidant and Antiproliferative Effects of Various Forms of Garlic and Ramsons. Molecules.

[40] Szychowski K.A., Rybczyńska-Tkaczyk K., Gaweł-Bȩben K., Ašwieca M., Kara M., Jakubczyk A., Matysiak M., Binduga U.E., Gmiński J., Gaweł-Bęben K. (2018). Characterization of Active Compounds of Different Garlic (*Allium sativum* L.) Cultivars. Pol. J. Food Nutr. Sci..

[41] Trekli M., Buttle D., Guesdon F. (2004). Anti-inflammatory Actions of Green Tea Catechins and Ligands of Peroxisome Proliferator-activated Receptors. Int. J. Exp. Pathol..

[42] Chen S., Shen X., Cheng S., Li P., Du J., Chang Y., Meng H. (2013). Evaluation of Garlic Cultivars for Polyphenolic Content and Antioxidant Properties. PLoS ONE.

[43] Aranaz P., Navarro-Herrera D., Zabala M., Miguéliz I., Romo-Hualde A., López-Yoldi M., Alfredo Martínez J., Vizmanos J.L., Milagro F.I., González-Navarro C.J. (2019). Phenolic Compounds Inhibit 3T3-L1 Adipogenesis Depending on the Stage of Differentiation and Their Binding Affinity to PPARγ. Molecules.

[44] Shi L., Lin Q., Li X., Nie Y., Sun S., Deng X., Wang L., Lu J., Tang Y., Luo F. (2017). Alliin, a Garlic Organosulfur Compound, Ameliorates Gut Inflammation through MAPK-NF-ΚB/AP-1/STAT-1 Inactivation and PPAR-γ Activation. Mol. Nutr. Food Res..

[45] Liu Y., Beyer A., Aebersold R. (2016). On the Dependency of Cellular Protein Levels on MRNA Abundance. Cell.

[46] Chen L., Hong J.-Y., So E., Hussin A.H., Cheng W.-F., Yang C.S. (1999). Decrease of Hepatic Catalase Level by Treatment with Diallyl Sulfide and Garlic Homogenates in Rats and Mice. J. Biochem. Mol. Toxicol..

[47] Mukherjee S., Banerjee S.K., Maulik M., Dinda A.K., Talwar K.K., Maulik S.K. (2003). Protection against Acute Adriamycin-Induced Cardiotoxicity by Garlic: Role of Endogenous Antioxidants and Inhibition of TNF-α Expression. BMC Pharmacol..

[48] Al-Brakati A. (2020). Protective Effect of Aged Garlic Extracts against Hepatotoxicity Induced by Ethephon in Wistar Albino Rat. Environ. Sci. Pollut. Res..

[49] Yang J., Song X., Feng Y., Liu N., Fu Z., Wu J., Li T., Chen H., Chen J., Chen C. (2020). Natural Ingredients-Derived Antioxidants Attenuate H_2_O_2_-Induced Oxidative Stress and Have Chondroprotective Effects on Human Osteoarthritic Chondrocytes via Keap1/Nrf2 Pathway. Free Radic. Biol. Med..

[50] Huang Y.-S., Xie N., Su Q., Su J., Huang C., Liao Q.-J. (2011). Diallyl Disulfide Inhibits the Proliferation of HT-29 Human Colon Cancer Cells by Inducing Differentially Expressed Genes. Mol. Med. Rep..

[51] Lemar K.M., Turner M.P., Lloyd D. (2002). Garlic (*Allium sativum*) as an Anti-Candida Agent: A Comparison of the Efficacy of Fresh Garlic and Freeze-Dried Extracts. J. Appl. Microbiol..

[52] Kaja S., Payne A.J., Naumchuk Y., Koulen P. (2017). Quantification of Lactate Dehydrogenase for Cell Viability Testing Using Cell Lines and Primary Cultured Astrocytes. Curr. Protoc. Toxicol..

[53] Szychowski K.A., Leja M.L., Kaminskyy D.V., Binduga U.E., Pinyazhko O.R., Lesyk R.B., Gmiński J. (2017). Study of Novel Anticancer 4-Thiazolidinone Derivatives. Chem. Biol. Interact..

[54] Nicholson D.W., Ali A., Thornberry N.A., Vaillancourt J.P., Ding C.K., Gallant M., Gareau Y., Griffin P.R., Labelle M., Lazebnik Y.A. (1995). Identification and Inhibition of the ICE/CED-3 Protease Necessary for Mammalian Apoptosis. Nature.

[55] Klimczak I., Małecka M., Szlachta M., Gliszczyńska-Świgło A. (2007). Effect of Storage on the Content of Polyphenols, Vitamin C and the Antioxidant Activity of Orange Juices. J. Food Compos. Anal..

