# Supplementary material for: Comparison of the Cytotoxic Mechanisms of Different Garlic (Allium sativum L.) Cultivars with the Crucial Involvement of Peroxisome Proliferator-Activated Receptor Gamma
Source: Int J Mol Sci. 2025 Jan 4;26(1):387. doi: 10.3390/ijms26010387 (PMC11720107; doi:10.3390/ijms26010387)

Supplementary data

Title: **Comparison of the cytotoxic mechanisms of different garlic (*Allium sativum* L.) cultivars with the crucial involvement of peroxisome proliferator-activated receptor gamma.**

Authors: **Urszula E. Binduga[1*], Aneta Kopeć[2], Joanna Skoczylas[2], Konrad A. Szychowski[3]**

[1]Department of Civilization Diseases and Regenerative Medicine, Medical College, University of Information Technology and Management in Rzeszow, st. Sucharskiego 2, 35-225 Rzeszow, Poland; ubinduga@wsiz.edu.pl

[2]Department of Human Nutrition and Dietetics, Faculty of Food Technology, Agricultural University of Krakow, st. Balicka 122, 30-149 Kraków, Poland; aneta.kopec@urk.edu.pl; joannaskoczylas7@gmail.com

[3]Department of Biotechnology and Cell Biology, Medical College, University of Information Technology and Management in Rzeszow, st. Sucharskiego 2, 35-225 Rzeszow, Poland; kszychowski@wsiz.edu.pl

*****Correspondence: ubinduga@wsiz.edu.pl

Example chromatograms from the analysis.

Garlic Harnaś cultivar, catechin – 6,126 – retention time

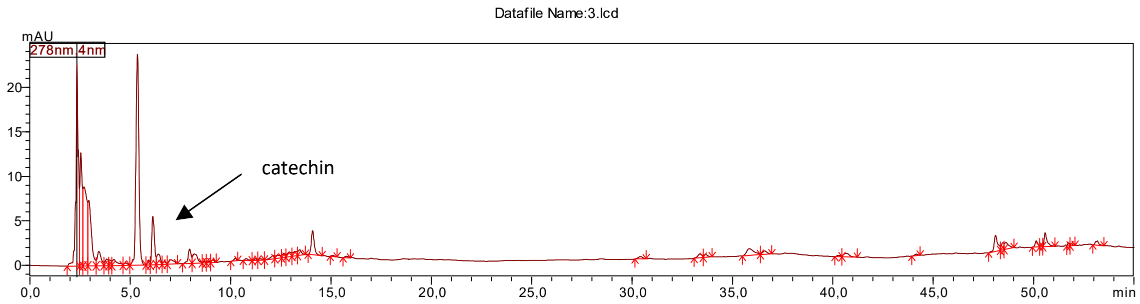

Garlic Ornak cultivar, catechin – 6,176 – retention time

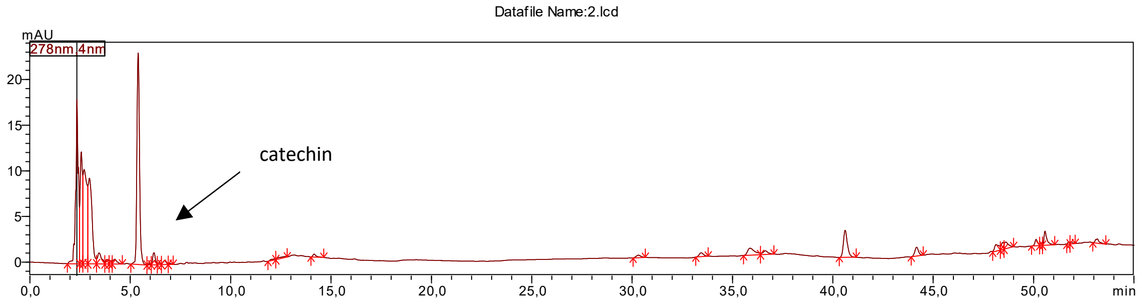

Garlic Violeta cultivar, naringin – 14,058 – retention time

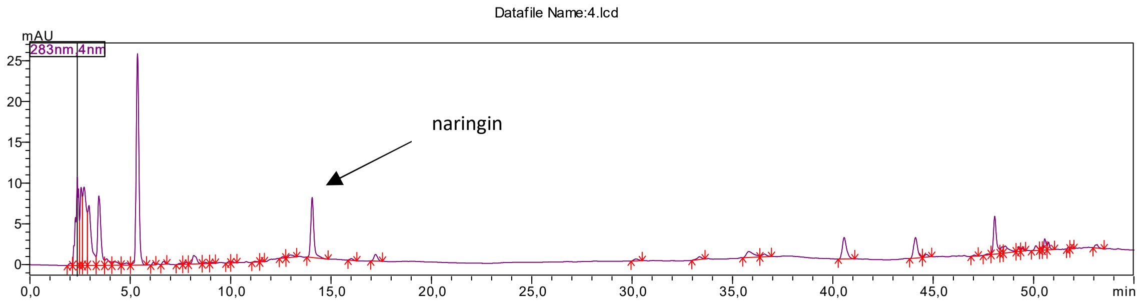

Garlic Morado cultivar, naringin – 14,254 – retention time

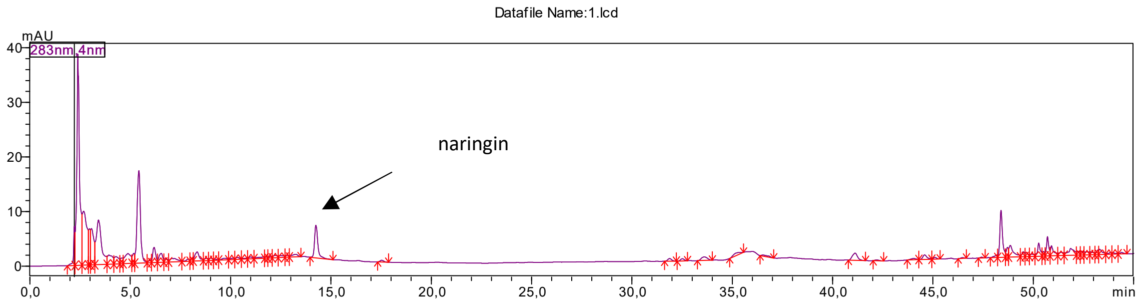

Supplement: Supplementary file 1 [file ijms-26-00387-s001.zip › ijms-3374996-supplementary.pdf]
